# Early History of Surgeons

**Published:** 1870-12

**Authors:** 


Early History of Surgeons.—There was a time when the profession of a surgeon and that of a barber went hand in hand. By a charter of Edward IV "the barbers practicing surgery" in the metropolis were legally incorporated as "The Company of the Barbers in London." Their authority extended to the right of examining all instruments and remedies, and of bringing actions against those who practiced ignorantly and illegally. The friends of competitive examination will also be glad to learn that even so far back as the first of Edward IV, there were examinations for surgical candidates by the masters of the company. But the first association had too much of the barber element in it. Many persons practiced surgery without its leave, and at last an association, which styled itself "The Surgeons of London." was formed at the beginning of Henry VIII's reign.

In the third year of that monarch it was enacted that no person within the city of London, or within seven miles of the same, should practice surgery unless he were "first examined and admitted by the Bishop of London or the Dean of St. Paul's," having "other expert persons in that faculty" as their assessors. In these associations it will be observed that surgery had thrown off the barber's basin; but the step was premature. The surgeons could not yet stand alone without the barbers, and in the thirty-second year of Henry VIII the Company of Barbers and the Company of Surgeons were united in one company, which still exists, with its hall, in Monkwell street, and its famous picture by Holbein, in which the "bluff King" may be seen giving their charter to the company. Thus the two trades—for neither could be called a profession—that of the basin and of the lancet—were inseparably joined together until the eighteenth year of George II, when the union was dissolved and the surgeons were constituted an independent company. It was not until the fortieth year of George III that the company passed into the hands of the Royal College of Surgeons.

We have been thus particular in tracing the early history of surgery in this country, and in showing how one of the noblest callings sprang from base beginnings, because we wish to point out the fact that it is learning and science which raise a mere trade like that of a barber, with his soap-suds and his basin, into an enlightened profession, which, while it alleviates suffering and confers the greatest blessing on the human race, is capable at the same time of satisfying the highest intellectual aspirations. So long as the surgeon went about blood-letting and tooth-drawing, now dressing a beard, and now "breathing a vein," he was little better than a barber, and was called a "barber surgeon." So long as he merely looked at the outward shape and fashion of the human frame, and now and then picked a hole in it or scraped its outer coat with a scalpel, he was merely a tradesman and quack, and his calling proportionately poor and mean. But when he passed from the outside of the wondrous house in which we all dwell, and penetrated into its innermost recesses; when he measured and surveyed it; when he undertook to renovate and restore it—nay, when he was bold enough to mutilate and deface parts of it in order that the rest might remain and live; when he learned, after long and laborious inquiry, after generations of thinkers, each of which, as it came, caught up and carried on the lamp of science when its predecessor dropped it; when he learned, we say, so many of the secrets of humanity, only to find how great his ignorance was and how much remained for him to learn—then his base trade had passed into a divine profession, and to the knowledge of a sage he had united the humility of a child. This we take to be very much the position of surgery at the present day. A true surgeon may feel proud as he looks back to the time of Henry VII, and the days of Barber Surgeons' Hall, but as he looks forward and feels how short life is, and how slow are the steps of science, he knows that, however great her victories over ignorance have been, she has many more pitched battles to win over her enemy before he shall be finally subdued and laid prostrate at her feet.—*London Telegraph.*

